# Relationship between Upper and Lower Body Strength and Basketball Shooting Performance

**DOI:** 10.3390/sports10100139

**Published:** 2022-09-20

**Authors:** Dimitrije Cabarkapa, Drake A. Eserhaut, Andrew C. Fry, Damjana V. Cabarkapa, Nicolas M. Philipp, Shay M. Whiting, Gabriel G. Downey

**Affiliations:** Jayhawk Athletic Performance Laboratory—Wu Tsai Human Performance Alliance, Department of Health, Sport, and Exercise Sciences, University of Kansas, Lawrence, KS 66045, USA

**Keywords:** free-throw, two-point, three-point, team sports, coaching, bench press, back squat

## Abstract

Strength is one of the key physiological performance attributes related to optimal on-court basketball performance. However, there is a lack of scientific literature studying how strength relates to shooting proficiency, as a key basketball skill capable of discriminating winning from losing game outcomes. Thus, the purpose of the present study was to examine the relationship between maximal upper and lower body strength and free-throw, two-point, and three-point shooting accuracy. Ten males and seven females performed bench press and back squat one repetition maximum (1RM) and basketball shooting testing during two laboratory visits. The shooting protocol consisted of five sets of 15 free-throw, two-point, and three-point shots performed in sequential order. Each set was separated by a 30 min rest interval to minimize the influence of fatigue. Each subject attempted 225 shots, combining for a total of 3825 shots. The average free-throw, two-point, and three-point shooting accuracy for men were 74.5 ± 11.9, 68.4 ± 9.9, and 53.3 ± 14.9%, and for women 79.2 ± 11.2, 65.5 ± 8.4, and 51.2 ± 15.3%, respectively. The average bench press and back squat 1RM for men was 88.2 ± 18.6 and 117.0 ± 21.2 kg, and for women, 40.6 ± 7.5 and 66.9 ± 9.9 kg, respectively. The findings of the present study revealed no significant relationships between maximal upper and lower body strength and basketball shooting performance for both male and female participants. Neither bench press nor back squat 1RM was a good predictor of free-throw, two-point, and three-point shooting performance.

## 1. Introduction

Basketball is one of the most popular international sports. It is a fast-paced game in which the only way to score points is by putting the ball through the basket. This can be achieved by attempting free-throw, two-point, and three-point shots. The importance of these types of shooting motions for securing the winning game outcome during both regular and post-season competitive periods has been well documented in the scientific literature [1,2,3,4,5]. Therefore, it is of critical importance for basketball players to know how to shoot a ball with a high level of proficiency, regardless of their playing position (e.g., guard, forward, center) and the level of competition.

In order to properly respond to on-court playing demands (e.g., jumping, sprinting, change-of-direction, shuffling), basketball players need to possess a unique blend of physiological performance attributes, including strength, power, speed, agility, and anaerobic and aerobic capacity [6,7,8,9]. In a recently published study, Cabarkapa et al. [10] found that lower body strength and power in male basketball players are positively related to post-collegiate playing opportunities, with greater values being associated with higher levels of professional play (*n* = 37). Hoffman et al. [11] found that playing time at the National Collegiate Athletic Association (NCAA) Division-I level of men’s basketball competition was positively correlated (r = 0.52–0.64) with back squat one repetition maximum (1RM), while the relationship with bench press 1RM was negligible (r = −0.04–0.14; *n* = 29). Vertical jump height, agility (i.e., *t*-test), and speed (i.e., 27 m sprint) were also good predictors of playing time [11]. While observing similar findings regarding the predictive ability of back squat 1RM (r = 0.74), Dawes et al. [6] found a significant positive association between bench press 1RM and playing time (r = 0.71) when examining male basketball players competing at the NCAA Division-II level. In addition, in a study conducted on professional male basketball players, Chaouachi et al. [12] found strong correlations between half-squat 1RM and sprinting performance over 5, 10, and 30 m. Greater half-squat 1RM values were associated with lower sprint times, further emphasizing the importance of lower body strength for eliciting improvements in physiological performance attributes related to on-court playing demands [12].

When examining physical determinants during an annual National Basketball Association (NBA) draft combine, drafted players outperformed non-drafted players in the three-quarter court sprint; however, no difference was found in the total number of repetitions performed for the bench press exercise at 83.9 kg [13]. This testing modality has also been shown to have a poor correlation with playing time, total games played, and minutes played per game at the NCAA Division-I level of men’s basketball competition [14]. On the other hand, Delextrat and Cohen [15] found that elite college male basketball players had significantly better bench press 1RM when compared to average-level players (+18.6%), alongside superior agility (i.e., *t*-test), and vertical jump height performance. The inability to observe significant findings in the aforementioned studies [13,14] may be attributed to the homogeneity of the sample, as these basketball players were elite athletes who may have already possessed the required levels of upper body strength. Moreover, it has been found that NCAA Division-I basketball players during their collegiate career are capable of attaining improvements in the bench press and squat 1RM by 24 and 32%, respectively [16].

In a recently conducted study, Pojskic et al. [17] examined the association between conditioning capacities and shooting performance within a cohort of professional basketball players. The authors found that jumping and anaerobic capacity (i.e., running-based anaerobic sprint test) were good determinants of shooting performance from long distances. In addition, no significant relationship was found between maximal aerobic capacity and basketball shooting performance [17]. When considering that these basketball players were elite athletes, these observations are not surprising as improvements in maximal aerobic capacity above the recommended ranges (i.e., 42–64 mL/kg/min) have not resulted in further performance enhancements [8,11]. 

Based on the previously mentioned findings, it is obvious that the majority of research has been directed toward examining differences in physiological performance attributes (e.g., strength, power, speed, agility) between different levels of play/experience as well as their association with playing time. Currently, there is a lack of scientific literature studying how physiological performance attributes relate to shooting accuracy, as a key basketball skill capable of discriminating winning from losing game outcomes [1,2,3,4,5]. Therefore, in order to bridge a gap in the scientific literature, the purpose of the present study was to examine the relationship between maximal upper and lower body strength (i.e., bench press and back squat 1RM) and basketball shooting performance (i.e., free-throw, two-point, and three-point) in both males and females.

## 2. Materials and Methods

### 2.1. Participants

Ten males (x ± SD; height = 182.6 ± 9.7 cm; body mass = 79.2 ± 13.9 kg; age = 25.6 ± 5.5 years; playing experience = 9.5 ± 4.1 years) and seven females (height = 174.5 ± 11.1 cm; body mass = 74.7 ± 11.8 kg; age = 24.4 ± 3.0 years; playing experience = 9.1 ± 2.3 years) volunteered to participate in this study. The following inclusion criteria were used: (i) currently competing or having previous playing experience at a high school, collegiate, or professional level of basketball competition; (ii) participating in basketball-specific training activities ≥2 times per week; (iii) ≥2 years of resistance training experience; (iv) participating in ≥2 resistance training sessions per week; (v) capable of making ≥50% of free-throw and two-point and ≥30% of three-point shooting attempts (this criterion was established based on feedback from a panel of experts consisting of former collegiate and professional basketball coaches and players). Participants with current and/or previous musculoskeletal injuries that could potentially impair lifting and/or shooting performance were excluded from participation. All testing procedures performed in this study were previously approved by the University of Kansas Institutional Review Board and all participants signed an informed consent document.

### 2.2. Procedures

The participants completed two laboratory visits 3–7 days apart. The first visit included familiarization with the testing design and procedures, and completion of a standardized warm-up protocol, basketball shooting qualification protocol, back squat 1RM, and bench press 1RM. The warm-up protocol consisted of a set of dynamic stretching exercises (e.g., butt-kicks, quad pulls, lateral lunges, A-skips, walking quad stretch) performed in sequential order. The shooting qualification protocol entailed the completion of 15 free-throw (4.57 m for men and women), 15 two-point (5.18 m for men and women), and 15 three-point shots (6.75 m for men and 6.33 m for women). Only participants that made ≥50% of free-throw and two-point and ≥30% of three-point shooting attempts were allowed to participate in the study. While 20 participants were initially recruited, three were excluded due to not meeting the aforementioned criteria. The 1RM testing procedures for both bench press and back squat resistance exercises followed guidelines established by the National Strength and Conditioning Association [18]. For 1RM bench press testing, participants were instructed to lie on the bench in a supine position (i.e., five-point body contact), grasp the barbell (Powerlifting Competition Bar—20 kg; Eleiko, Halmstad, Sweden) with a closed pronated grip shoulder-width apart, and perform repetitions with the barbell positioned over the chest with maximal effort. For 1RM back squat testing, participants were instructed to grasp the barbell with a closed pronated grip and place it on the upper trapezius maximus at the base of the neck, hold the chest up and out, tilt the head slightly up, take one to two steps backward, position the feet shoulder-width apart, and perform repetitions with maximal effort. For both exercises, the participants were asked to perform a set of 5–10 repetitions of self-selected light-to-moderate weights, followed by two heavier sets of 3–5 repetitions. In 2–3 min increments, the weight was increased by 5–10% after each successfully completed lift until the maximal amount of weight that the participant is capable of lifting is reached [18]. 

**Figure 1:**
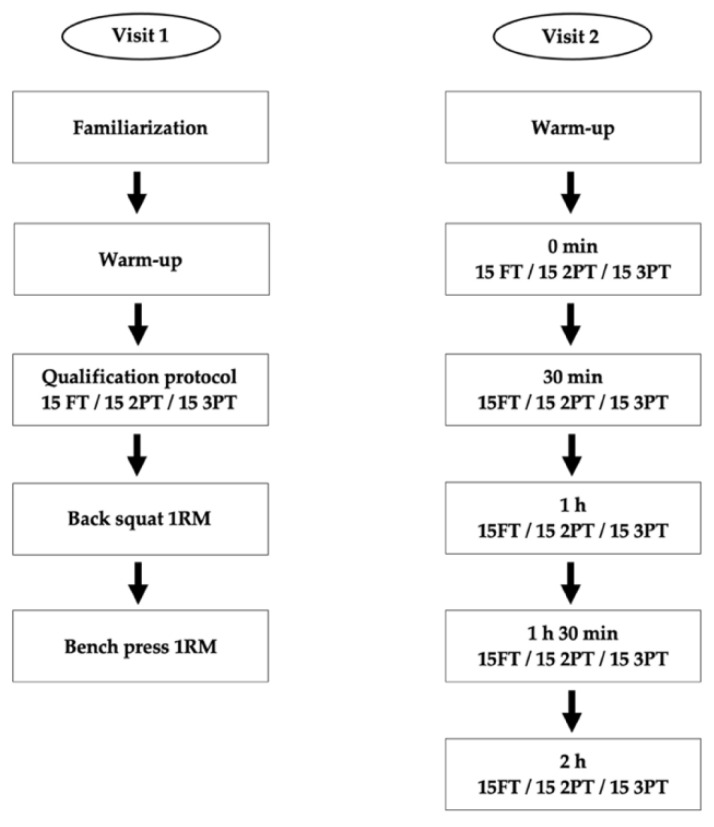
Graphical representation of the testing procedures. 1RM—one repetition maximum; FT—free-throw; 2PT—two-point; 3PT—three-point.

The second visit included the completion of a standardized warm-up protocol followed by five sets of 15 free-throw, 15 two-point, and 15 three-point shots performed in sequential order. Each set was separated by a 30 min rest interval to minimize the influence of fatigue and optimize recovery. Each subject attempted 225 shots, combining for a total of 3825 shots. A research assistant was present throughout the full testing procedure to help with rebounding and passing tasks. To eliminate any kind of possible distractions, participants individually performed all testing procedures. Basketball goal height (3.05 m) and size corresponded to men’s (0.75 m, 0.62 kg; Wilson Evolution Indoor, Chicago, IL, USA) and women’s (0.72 m, 0.57 kg; Wilson Evolution Indoor, Chicago, IL, USA) basketball international regulations standards. The graphical representation of the testing procedures is shown in Figure 1. 

### 2.3. Statistical Analysis

Descriptive statistics, including means and standard deviations (x ± SD), were calculated for each dependent variable. Free-throw, two-point, and three-point shooting performance were measured as a percentage of shots made during the second laboratory visit. After meeting the assumptions, linear regression analysis was used to examine the relationship between maximal upper and lower body strength (i.e., bench press and back squat 1RM) and shooting performance (i.e., free-throw, two-point, and three-point shooting accuracy), separately for men and women due to differences in ball size, three-point shot distance, and physiological characteristics. Post hoc power analysis and Cohen’s *f*^2^ effect sizes were calculated via G*Power software (Version 3.1; Heinrich Heine University, Dusseldorf, Germany). Statistical significance was set a priori to *p* < 0.05. Linear regression analyses were completed with SPSS (Version 26.0; IBM Corp., Armonk, NY, USA).

## 3. Results

The average free-throw, two-point, and three-point shooting accuracy for men was 74.5 ± 11.9, 68.4 ± 9.9, and 53.3 ± 14.9%, and for women 79.2 ± 11.2, 65.5 ± 8.4, and 51.2 ± 15.3%, respectively. The average bench press and back squat 1RM for men was 88.2 ± 18.6 and 117.0 ± 21.2 kg, and for women, 40.6 ± 7.5 and 66.9 ± 9.9 kg, respectively. 

**Figure 2:**
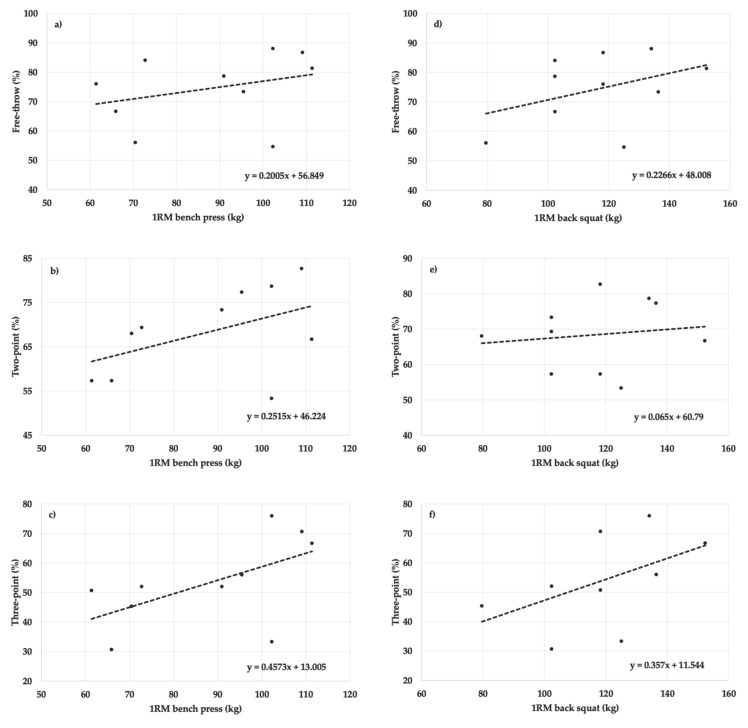
Scatter plots for the relationship between: (**a**) 1RM bench press and free-throw shooting performance, (**b**) 1RM bench press and two-point shooting performance, (**c**) 1RM bench press and three-point shooting performance, (**d**) 1RM back squat and free-throw shooting performance, (**e**) 1RM back squat and two-point shooting performance, and (**f**) 1RM back squat and three-point shooting performance for men.

No statistically significant relationships were found between bench press 1RM and free-throw (r = 0.316, R^2^ = 0.100, F_[1,8]_ = 0.890, *p* = 0.373, *f*^2^ = 0.111, power = 0.158), two-point (r = 0.477, R^2^ = 0.228, F_[1,8]_ = 2.358, *p* = 0.163, *f*^2^ = 0.295, power = 0.328), and three-point shooting performances (r = 0.580, R^2^ = 0.337, F_[1,8]_ = 4.062, *p* = 0.079, *f*^2^ = 0.508, power = 0.509), and back squat 1RM and free-throw (r = 0.401, R^2^ = 0.161, F_[1,8]_ = 1.536, *p* = 0.250, *f*^2^ = 0.192, power = 0.231), two-point (r = 0.138, R^2^ = 0.019, F_[1,8]_ = 0.156, *p* = 0.703, *f*^2^ = 0.019, power = 0.068), and three-point shooting performances (r = 0.508, R^2^ = 0.259, F_[1,8]_ = 2.790, *p* = 0.133, *f*^2^ = 0.349, power = 0.377) for men. See Figure 2.

**Figure 3:**
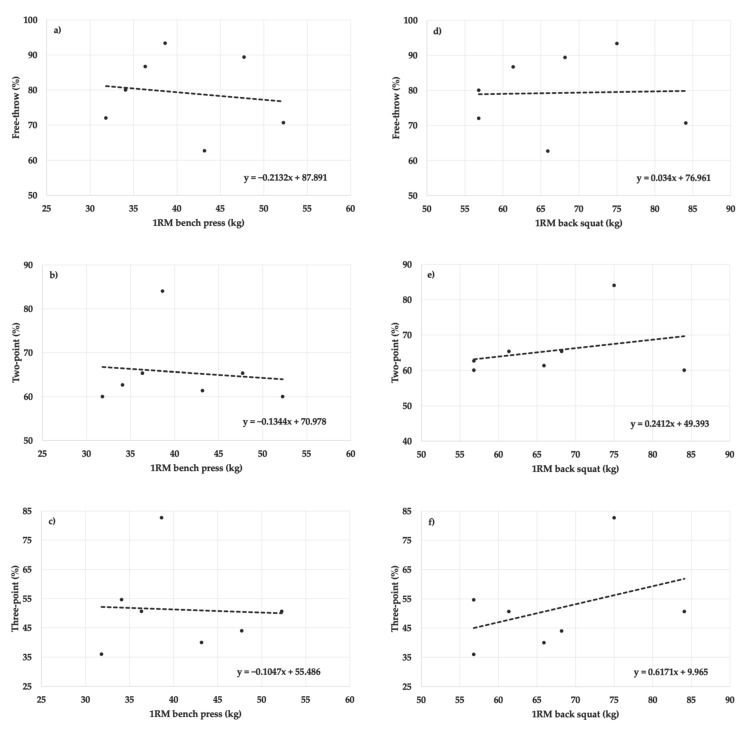
Scatter plots for the relationship between: (**a**) 1RM bench press and free-throw shooting performance, (**b**) 1RM bench press and two-point shooting performance, (**c**) 1RM bench press and three-point shooting performance, (**d**) 1RM back squat and free-throw shooting performance, (**e**) 1RM back squat and two-point shooting performance, and (**f**) 1RM back squat and three-point shooting performance for women.

No statistically significant relationships were found between bench press 1RM and free-throw (r = 0.142, R^2^ = 0.020, F_[1,5]_ = 0.103, *p* = 0.762, *f*^2^ = 0.020, power = 0.061), two-point (r = 0.119, R^2^ = 0.014, F_[1,5]_ = 0.072, *p* = 0.800, *f*^2^ = 0.014, power = 0.058), and three-point shooting performances (r = 0.051, R^2^ = 0.003, F_[1,5]_ = 0.013, *p* = 0.914, *f*^2^ = 0.004, power = 0.052), and back squat 1RM and free-throw (r = 0.030, R^2^ = 0.001, F_[1,5]_ = 0.005, *p* = 0.949, *f*^2^ = 0.001, power = 0.050), two-point (r = 0.285, R^2^ = 0.081, F_[1,5]_ = 0.442, *p* = 0.536, *f*^2^ = 0.088, power = 0.099), and three-point shooting performances (r = 0.402, R^2^ = 0.161, F_[1,5]_ = 0.962, *p* = 0.317, *f*^2^ = 0.192, power = 0.158) for women. See Figure 3.

## 4. Discussion

The findings of the present study revealed no significant relationships between maximal upper or lower body strength and basketball shooting performance for both male and female participants. Neither bench press nor back squat 1RM was a good predictor of free-throw, two-point, and three-point shooting accuracy. To the best of our knowledge, this is the first study that examined the relationship between this physiological performance attribute (i.e., strength) and basketball on-court shooting performance.

A considerable amount of scientific literature has documented the importance of lower body strength as one of the key performance characteristics that basketball players need to possess [6,8,10,11,12]. Back squat 1RM was poorly correlated with playing time during the season when basketball players were not involved in any kind of off-season strength training program, while the same performance attribute become the strongest predictor of playing time in subsequent years when athletes were exposed to a structured and supervised strength training program [11]. Therefore, it is understandable why the squat and its variations are one of the most frequently implemented resistance exercises by strength and conditioning practitioners at the NBA level of basketball competition [19]. The previously mentioned research studies seem to contradict the findings of the present study, as no significant relationships were observed between back squat 1RM and free-throw, two-point, and three-point shooting accuracy. However, interpreting the results without considering the participants’ resistance training history/experience may be misleading. The back squat 1RM magnitudes observed in the present investigation were greater than previously reported ranges for untrained men and women and lower than previously reported ranges for professional and collegiate basketball players, which is expected considering that the cohort of participants examined in the present study were resistance-trained individuals with previous basketball playing experience [8,20,21,22]. Therefore, as a possible explanation for the non-statistically significant relationship, we may assume that the participants already possessed the level of lower body strength needed to successfully execute these types of shooting motions.

Upper body strength is another physical performance attribute required by basketball players when competing, to create and defend space on the court [8]. The bench press 1RM has been widely used as a measure of maximal upper body strength among basketball players competing at various levels of competition [6,11,14,15]. In addition, the total number of repetitions performed for the bench press exercise (i.e., 83.9 kg) has been a part of the NBA draft combine testing procedures for decades [23]. However, unlike back squat 1RM, previous research has reported mixed findings between bench press 1RM and on-court playing performance. Lockie et al. [14] and Hoffman et al. [11] found no significant relationships in male basketball players between bench press 1RM and playing time at the NCAA Division-I competitive level, while Dawes et al. [6] found a strong positive relationship between the same variables at the NCAA Division-II level of competition. Moreover, Cui et al. [13] found no significant difference in upper body strength between drafted and undrafted NBA players, while Teramoto et al. [23] found that the same testing modality was valuable in predicting the future performance of players. The findings of the present study add to the complexity and ambiguity of the previously mentioned findings examining the association between upper body strength and basketball on-court performance, as no significant relationships were observed between bench press 1RM and free-throw, two-point, and three-point shooting accuracy. The bench press 1RM magnitudes for male participants were well within previously established ranges for professional and collegiate basketball players [8,9], while female participants demonstrated slightly lower levels of upper body strength than previously reported for the NCAA Division-I athletes [24]. Thus, similar to the observations regarding lower body strength, the non-statistically significant relationship between upper body strength and basketball shooting performance may be explained by the assumption that the participants already possessed the level of upper body strength needed to successfully execute these types of shooting motions.

Overall, it is important to note that the findings of the present study do not suggest that the value of upper and lower body strength should be diminished, but rather imply that there might be other factors that influence optimal shooting performance, such as the kinematics of basketball shooting form. Knudson [25] indicated that when teaching/coaching shooting form, a player should be instructed to minimize horizontal motion and maintain near-vertical trunk alignment as these kinematics adjustments may elicit improvements in jump shooting performance. Cabarkapa et al. [26] found that greater elbow flexion and higher elbow positioning during the preparatory phase of the two-point shooting motion and greater vertical jump displacement at the time point of the ball release for three-point shooting motion may serve as beneficial coaching cues directed towards the improvement of shooting form. In addition, it has been found that lower elbow positioning achieved by greater flexion in the knees, hips, and ankles and minimizing lateral elbow deviation during the preparatory phase of the shooting motion were key kinematics characteristics distinguishing between proficient and non-proficient free-throw shooters [27,28].

Further research is warranted to examine whether advancement in basketball shooting performance is primarily influenced by attaining greater levels of upper and lower body strength and/or improvements in shooting form kinematics as well as their respective contributions. In addition, future research needs to examine differences in upper and lower body strength between various competitive levels and age ranges, and determine the threshold after which further strength gains do not translate into improvements in on-court basketball performance. While offering additional insight into the relationship between physical performance characteristics (i.e., maximal upper and lower body strength) and basketball shooting capabilities, this study is not without limitations. The sample of participants that volunteered to participate in the present investigation is homogenous (e.g., no untrained and/or highly trained athletes) and could have been larger in size. In addition, all shooting procedures were conducted in a controlled laboratory environment. It is possible that the presence of a defender [29,30], nutrition status [31], and/or completion of shooting protocols in non-fatiguing conditions [32] could have influenced the findings of the present study, as it did not precisely mimic the regular competitive environment.

## 5. Conclusions

The findings of the present study revealed no significant relationships between maximal upper and lower body strength and basketball shooting performance for both male and female participants. Neither bench press nor back squat 1RM was a good predictor of free-throw, two-point, and three-point shooting accuracy. However, it is important to note that the findings of the present study do not diminish the value of upper and lower body strength as one of the key physiological characteristics required for optimal basketball on-court performance, but rather imply that there might be other factors that influence optimal shooting performance that need to be considered and studied in future.

## Data Availability

The data presented in this study are available on request from the corresponding author.

## References

[1] Csataljay G., O’Donohue P., Huges M., Dancs H. (2009). Performance indicators that distinguish winning and losing teams in basketball. Int. J. Perform. Anal..

[2] Csataljay G., James N., Hughes M., Dancs H. (2012). Performance difference between winning and losing basketball teams during close, balanced and unbalanced quarters. J. Hum. Sport Exerc..

[3] Gomez M.A., Lorenzo A., Sampaio J., Ibanez S.J., Ortega E. (2008). Game-related statistics that discriminated winning and losing teams from the Spanish men’s professional basketball teams. Coll. Antropol..

[4] Lorenzo A., Gomez M.A., Ortega E., Ibanez S.J., Sampaio J. (2010). Game related statistics which discriminate between winning and losing under-16 male basketball games. J. Sport Sci. Med..

[5] Trninic S., Dizdar D., Luksic E. (2002). Difference between winning and defeated top quality basketball teams in final tournaments of European club championship. Coll. Antropol..

[6] Dawes J.J., Spiteri T. (2016). Relationship between pre-season testing performance and playing time among NCAA D-II basketball players. Sport Exerc. Med..

[7] Ostojic S.M., Mazic S., Dikic N. (2006). Profiling in basketball: Physical and physiological characteristics of elite players. J. Strength Cond. Res..

[8] Morrison M., Martin D.T., Talpey S., Scanlan A.T., Delaney J., Halson S.L., Weakley J. (2022). A systematic review on fitness testing in adult male basketball players: Tests adopted, characteristics reported and recommendations for practice. Sports Med..

[9] Latin R.W., Berg K., Baechle T. (1994). Physical and performance characteristics of NCAA Division-I male basketball players. J. Strength Cond. Res..

[10] Cabarkapa D., Fry A.C., Lane M.T., Hudy A., Dietz P.R., Cain G.J., Andre M.J. (2020). The importance of lower body strength and power for future success in professional men’s basketball. Sport Sci. Health.

[11] Hoffman J.R., Tenenbaum G., Maresh C.M., Kraemer W.J. (1996). Relationship between athletic performance tests and playing time in elite college basketball players. J. Strength Cond. Res..

[12] Chaouachi A., Brughelli M., Chamari K., Levin G.T., Abdelkrim N.B., Laurencelle L., Castagna C. (2009). Lower limb maximal dynamic strength and agility determinants in elite basketball players. J. Strength Cond. Res..

[13] Cui Y., Liu F., Bao D., Liu H., Zhang S., Gómez M.Á. (2019). Key anthropometric and physical determinants for different playing positions during National Basketball Association draft combine test. Front. Psychol..

[14] Lockie R.G., Beljic A., Ducheny S.C., Kammerer J.D., Dawes J.J. (2020). Relationships between playing time and selected NBA combine test performance in Division-I mid-major basketball players. Int. J. Exerc. Sci..

[15] Delextrat A., Cohen D. (2008). Physiological testing of basketball players: Toward a standard evaluation of anaerobic fitness. J. Strength Cond. Res..

[16] Hunter G.R., Hilyer J., Forster M.A. (1993). Changes in fitness during 4 years of intercollegiate basketball. J. Strength Cond. Res..

[17] Pojskic H., Sisic N., Separovic V., Sekulic D. (2018). Association between conditioning capacities and shooting performance in professional basketball players: An analysis of stationary and dynamic shooting skills. J. Strength Cond. Res..

[18] Haff G.G., Triplett N.T. (2015). Essentials of Strength Training and Conditioning.

[19] Simenz C.J., Dugan C.A., Ebben W.P. (2005). Strength and conditioning practices of National Basketball Association strength and conditioning coaches. J. Strength Cond. Res..

[20] McCurdy K., Langford G.A., Cline A.L., Doscher M., Hoff R. (2004). The reliability of 1-and 3RM tests of unilateral strength in trained and untrained men and women. J. Sport Sci. Med..

[21] Rice P.E., Goodman C.L., Capps C.R., Triplett N.T., Erickson T.M., McBride J.M. (2017). Force–and power–time curve comparison during jumping between strength-matched male and female basketball players. Eur. J. Sport Sci..

[22] Witmer C.A., Davis S.E., Moir G.L. (2010). The acute effects of back squats on vertical jump performance in men and women. J. Sports Sci. Med..

[23] Teramoto M., Cross C.L., Rieger R.H., Maak T.G., Willick S.E. (2018). Predictive validity of national basketball association draft combine on future performance. J. Strength Cond. Res..

[24] Burnham T.R., Ruud J.D., McGowan R. (2010). Bench press training program with attached chains for female volleyball and basketball athletes. Percept. Mot. Skills.

[25] Knudson D. (1993). Biomechanics of the basketball jump shot—Six key teaching points. J. Phys. Educ. Recreat. Dance.

[26] Cabarkapa D., Fry A.C., Cabarkapa D.V., Myers C.A., Jones G.T., Deane M.A. (2021). Kinetic and kinematic characteristics of proficient and non-proficient 2-point and 3-point basketball shooters. Sports.

[27] Cabarkapa D., Fry A.C., Carlson K.M., Poggio J.P., Deane M.A. (2021). Key kinematic components for optimal basketball free throw shooting performance. Cent. Eur. J. Sport Sci. Med..

[28] Cabarkapa D., Fry A.C., Poggio J.P., Deane M.A. (2021). Kinematic differences between proficient and non-proficient free throw shooters–video analysis. J. Appl. Sports Sci..

[29] Esteves P.T., Arede J., Travassos B., Dicks M. (2021). Gaze and shoot: Examining the effects of player height and attacker-defender interpersonal distances on gaze behavior and shooting accuracy of elite basketball players. Rev. Psicol. Deporte.

[30] Gorman A.D., Maloney M.A. (2016). Representative design: Does the addition of a defender change the execution of a basketball shot?. Psychol. Sport Exerc..

[31] Cabarkapa D., Fry A.C., Deane M.A., Akers J.D. (2020). The relationship between breakfast consumption and basketball shooting performance. Facta Univ. Ser. Phys. Educ. Sport.

[32] Padulo J., Nikolaidis P.T., Cular D., Dello Iacono A., Vando S., Galasso M., Storto D.L., Ardigo L.P. (2018). The effect of heart rate on jump-shot accuracy of adolescent basketball players. Front. Physiol..

